# No Evidence of Pathogenic Involvement of Cathelicidins in Patient Cohorts and Mouse Models of Lupus and Arthritis

**DOI:** 10.1371/journal.pone.0115474

**Published:** 2014-12-23

**Authors:** D. Kienhöfer, J. Hahn, I. Schubert, C. Reinwald, N. Ipseiz, S. C. Lang, È. Bosch Borràs, K. Amann, C. Sjöwall, A. E. Barron, A. J. Hueber, B. Agerberth, G. Schett, M. H. Hoffmann

**Affiliations:** 1 Department of Internal Medicine 3, Universitätsklinikum Erlangen, Erlangen, Germany; 2 Universitat Autònoma de Barcelona, Barcelona, Spain; 3 Department of Nephropathology, Universitätsklinikum Erlangen, Erlangen, Germany; 4 Rheumatology/AIR, Department of Clinical and Experimental medicine, Linköping university, Linköping, Sweden; 5 Department of Bioengineering, Stanford University, Stanford, CA, United States of America; 6 Division of Clinical Microbiology, F68, Department of Laboratory Medicine, Karolinska University, Huddinge, Stockholm, Sweden; INSERM-Université Paris-Sud, France

## Abstract

Apart from their role in the immune defence against pathogens evidence of a role of antimicrobial peptides (AMPs) in autoimmune diseases has accumulated in the past years. The aim of this project was to examine the functional impact of the human cathelicidin LL-37 and the mouse cathelicidin-related AMP (CRAMP) on the pathogenesis of lupus and arthritis. Serum LL-37 and anti-LL-37 levels were measured by ELISA in healthy donors and patients with Systemic Lupus Erythematosus (SLE) and Rheumatoid arthritis (RA). Pristane-induced lupus was induced in female wild type (WT) and cathelicidin-deficient (CRAMP^−/−^) mice. Serum levels of anti-Sm/RNP, anti-dsDNA, and anti-histone were determined via ELISA, cytokines in sera and peritoneal lavages were measured via Multiplex. Expression of Interferon I stimulated genes (ISG) was determined by real-time PCR. Collagen-induced arthritis (CIA) was induced in male WT and CRAMP^−/−^ mice and arthritis severity was visually scored and analysed histomorphometrically by OsteoMeasure software. Serum levels of anti-LL-37 were higher in SLE-patients compared to healthy donors or patients with RA. However, no correlation to markers of disease activity or organ involvement was observed. No significant differences of autoantibody or cytokine/chemokine levels, or of expression of ISGs were observed between WT and CRAMP^−/−^ mice after pristane-injection. Furthermore, lung and kidney pathology did not differ in the absence of CRAMP. Incidence and severity of CIA and histological parameters (inflammation, cartilage degradation, and bone erosion) were not different in WT and CRAMP^−/−^ mice. Although cathelicidins are upregulated in mouse models of lupus and arthritis, cathelicidin-deficiency did not persistently affect the diseases. Also in patients with SLE, autoantibodies against cathelicidins did not correlate with disease manifestation. Reactivity against cathelicidins in lupus and arthritis could thus be an epiphenomenon caused by extensive overexpression in blood and affected tissues. In addition, other cationic AMPs could functionally compensate for the deficiency of cathelicidins.

## Introduction

Apart from their role in anti-microbial defence, anti-microbial peptides (AMPs) such as the human cathelicidin LL-37 possess potent immunomodulatory properties and have recently also been implicated in the pathogenesis of autoimmune diseases [1]–[4]. In sera of patients with Systemic Lupus Erythematosus (SLE), immune complexes of AMPs and self-DNA derived from neutrophil extracellular traps (NETs) were reported to trigger activation of Toll-like receptor (TLR) 9. Furthermore, SLE-patients were found to develop autoantibodies (autoAbs) to both self-DNA and AMPs [2], [3].

Patients with SLE [5], [6] and a subset of RA patients [7] display a type I interferon (IFN) signature in their peripheral blood mononuclear cells. Given their reported role in SLE, AMPs may also stimulate TLR-pathways in other autoimmune diseases characterized by reactivity to nucleic acids, such as arthritis. In a previous study, we observed overexpression of LL-37 and its rat homologue rCRAMP in arthritic joints of patients with RA and of rats, respectively. In rat pristane-induced arthritis, the increased expression of rCRAMP coincided with the development of anti-rCRAMP autoAbs [8].

We have now continued to further investigate the functional importance of cathelicidins, using sera from patient cohorts with SLE and RA and cathelicidin-deficient mice. Although we detected autoAbs to cathelicidins in humans and in mice with lupus, they were not linked to disease activity or severity. Furthermore, in mouse models of arthritis and inducible lupus, cathelicidin-deficient mice developed a disease comparable in severity to wild type (WT) animals. Our results therefore do not support previous reports about an indispensable role of cathelicidins in the pathogenesis of lupus and arthritis.

## Methods

### Animals

C57BL/6 mice deficient of CRAMP (CRAMP^−/−^ mice) were created as described [9] and kindly provided together with WT littermates by the groups of Lennart Lindbom, Karolinska Institute, Sweden, and Oliver Söhnlein, Technical University of Munich, Germany. Mice were bred and maintained at the animal facility of the University of Erlangen-Nuremberg. Experiments were performed on mice frequency-matched for age and sex, and evaluated with blinded identity.

### Patients

In this study, 185 patients with SLE (mean age 44 years, range 18–77), 92 patients with RA (mean age 49 years, range 20–107), and 67 sex-matched healthy controls (mean age 39 years, range 25–72) from cohorts of the university hospitals Erlangen and Linköping (longitudinal samples with clinical data for SLE) were included (S1 Table). All patients fulfilled at least 4 of the classification criteria for SLE [10] and RA [11], respectively, and gave written informed consent. Disease activity of the SLE patients was registered according to the SLE disease activity index 2000 (SLEDAI). In the longitudinal follow up examination, patients with more than 4 visits (follow up for >1 year) were considered for blood analysis and recording of disease activity parameters (SLEDAI). All experiments were approved by the local ethical committees (“Linköping university Ethical Review Board”, permit number M75-08, “Ethics committee of the university Erlangen”, permit number Re.-No. 3982 and Re.-No. 187_12B).

### Experimental lupus and arthritis

Pristane-induced lupus (PIL) was induced by a single intraperitoneal (i.p.) injection of 500 µl pristane (2, 6, 10, 14-tetramethyl-pentadecane; Sigma-Aldrich) in female C57BL/6 CRAMP^−/−^ and corresponding WT mice with an age of 8–12 weeks and followed over a period of 6 months. A limited number of mice were sacrificed after 14 days to enable histopathological analysis of the lungs and cytokine measurement of peritoneal lavages.

Collagen-induced arthritis (CIA) was induced in 8–12 weeks old male C57BL/6 CRAMP^−/−^ and WT mice by intradermal (i.d.) injection of 100 µg chicken collagen type II (Sigma-Aldrich) emulsified in 100 µl Incomplete Freund’s Adjuvant (IFA) supplemented with 5 mg/ml M*ycobacterium tuberculosis* (both from Sigma-Aldrich) at the base of the tail, followed by an i.d. booster injection of 100 µg CII/100 µl IFA 21 days later.

The severity of arthritis was graded visually by assessing the level of swelling in each paw, including the tarsus or carpus joints. The following scoring system was used: 0, no erythema/swelling; 1, erythema and swelling in up to two joints; 2, erythema and swelling in more than two joints or slight swelling of the ankle; 3, marked swelling of the ankle; 4, substantial swelling of the whole paw including toes/fingers. The maximum score per mouse was 16.

All experiments were approved by the local ethical committee (“Regierung von Mittelfranken”, permit number 54.2532.1-32/12).

### Analysis of organ involvement

To evaluate kidney involvement in PIL, proteinuria was determined with semiquantitative urine testing strips (Albustix, Bayer) using midstream urine. For assessing glomerular immune and complement deposits, mice were sacrificed and perfused with PBS to minimize background. One kidney/mouse was embedded in OCT Tissue-Tek compound (Sakura, Alphen aan den Rijn), snap-frozen, and stored at −80°C until use. 5 µm cryo-sections were fixed with acetone and stained with FITC-labelled antibodies to murine complement factor C3 (Cedarlane) and IgG (BioLegend), respectively. Glomerular deposits were scored by two blinded individual scorers by immunofluorescence microscopy on a Nikon Eclipse 80i microscope, utilizing a semiquantitative scoring system as described [12]. The other kidney was fixated in Roti-Histofix (Carl Roth) and embedded in paraffin. A Periodic acid Schiff (PAS) staining was prepared of 5 µm-sections, which were analyzed with a Zeiss AxioLab.A1 microscope.

### Analysis of CRAMP expression

Pristane was injected i.p. in WT and CRAMP^−/−^ mice and cells were collected via peritoneal lavage with PBS or after erythrocyte lysis from peripheral blood at day 0 and 7 after injection. Cells were stained extracellularly with anti-CD11b-eFluor450 (eBioscience), anti-Ly6C-APC (BioLegend), anti-CD115-PE (BioLegend), and anti-Ly6G-APC (BioLegend) to determine cell types. Intracellular CRAMP staining was performed with an anti-CRAMP (Innovagen) antibody labelled with an Alexa Fluor 488 antibody labelling kit (LifeTechnologies). Cells were then analyzed by cytofluorometry on a Gallios machine (Beckman Coulter).

### Determination of Autoantibodies and Serum Cytokine Levels

For detection of murine autoAbs by ELISA, microtiter plates were coated overnight with 50 µg/ml histone from calf thymus (Roche Diagnostics), 1 µg/ml Sm/RNP (GenWay Biotech), or precoated with 20 µg/ml poly-L-Lysine (Sigma-Aldrich) before adding 20 µg/ml calf thymus DNA (Sigma-Aldrich), respectively. PIL-sera were added at a 1∶100 dilution in PBS/2% FCS. Bound IgG was detected with 0.125 µg/ml horseradish peroxidase-conjugated goat anti-mouse IgG (Southern Biotech) and substrate solution (eBioScience).

For detection of anti-LL-37, microtiter plates were coated with 1 µg/ml LL-37 (Innovagen) for 2 hours at room temperature. Sera were diluted 1∶500 and incubated for 90 min at room temperature. After several washing steps with PBS/0.05% Tween, bound anti-LL-37 was detected using 0.02 µg/ml horseradish peroxidase-conjugated goat anti-human IgG antibody (Southern Biotech) and substrate solution (eBioScience). Detection was carried out with a Tecan Infinite F200 Pro microplate reader (TECAN) at 450 nm/620 nm. Cut-off for positivity was set at mean OD-index of normal healthy donor (NHD) sera+3 SD. Human and murine cytokines/chemokines in sera/plasma and peritoneal lavages were analyzed by multiplex bead technology (eBioscience) and quantified by cytofluorometry on a Gallios machine (Beckman Coulter). Serum levels of LL-37 were measured using a commercially available kit (HyCult). Cut-off for positivity was set at mean concentration in NHD sera+2 SD.

### Real time quantitative PCR

RNA from serum from WT and CRAMP^−/−^ mice collected at day 0, 30, and 180 after pristane injection was purified using the Direct-zol RNA MiniPrep Kit (Zymo Research). RNA was reverse-transcribed with a high-capacity cDNA Reverse Transcription Kit (Applied Biosystems). RT- PCR was performed using the qPCR Mastermix Plus for SYBR Green I (Eurogentec) on a QuantStudio6Flex (Applied Biosystems). Data were normalized to β-Actin by the ΔΔCt method. PCR primers were synthesized by Invitrogen: β-actin: TGTCCACCTTCCAGCAGATGT (for), AGCTCAGTAACAGTCCGCCTAGATGT (rev); MX-1: GATCCG ACTTCACTTCCAGATGG (for), CATCTCAGTGGTAGTCAACCC (rev); IP-10/CXCL10: TCCTGCTGGGTCTGAGTGGGAC (for), CGTGGCAATGATCT CAACACGTCG (rev); IRF-7: TGATGCCGGGGACCTCTTGCT (for), CTGCGCTCGG TGAGAGCTGG (rev).

### Complex formation

Plasmacytoid dendritic cells (pDCs) were isolated from leukocyte suspensions enriched from human blood in leukocyte reduction chambers as described [13] or from erylysed mouse whole blood by MACS using the according isolation kits (Miltenyi Biotec). 3×10^5^ isolated pDCs or 2,5×10^6^ whole blood cells were then incubated in complete DMEM medium for 24 h at 37°C with 0,5 µM Type A CpG 2216 (InvivoGen), 1 µg/ml R848 (Resiquimod, InvivoGen), 10 µg/ml genomic DNA (Novagen), 1 µg/ml total RNA isolated from human cell cultures or murine splenocytes using Trizol solution (Sigma-Aldrich), 50 µg/ml CRAMP/LL37 (both from Innovagen), or CRAMP/LL-37 complexed to DNA or RNA, respectively, as described [1], [4]. Type I IFN in supernatants was measured by a luciferase assay using HEK293 IFN reporter cells as described [14] or by multiplex bead technology (eBioscience).

### Histological and histomorphometrical analysis

Paws from naïve mice and mice 37 days after the first immunization were fixed overnight in 4% formalin, decalcified with EDTA, and embedded in paraffin. Paraffin sections were stained with haematoxylin&eosin (H&E), toluidine blue, and tartrate-resistant acid phosphatase (TRAP) for assessment of inflammation, proteoglycan loss in cartilage, and osteoclasts, respectively. Analysis was performed on a Nikon microscope equipped with a digital analysis system (OsteoMeasure). The areas of inflammation and bone erosions in all digital, carpal and tarsal joints were evaluated in H&E- and TRAP-stained sections as the sum of the areas of inflammation and erosions, respectively. For determination of cartilage proteoglycan loss the areas of total cartilage and destained cartilage were measured in sections stained with toluidine blue.

### Analysis of NETosis

IgG was purified from naïve mice and mice 180 days after i.p, injection of pristane using Dynabeads Protein G (Life technologies). Concentrations were measured at a NanoDrop machine (Thermo Fisher). Pooled heparinized whole blood from 5 mice was incubated for 3 h at 37°C/5% CO_2_ with either PBS, 100 nM PMA, 1 µg/ml or 0.1 µg/ml polyclonal anti-mouse CRAMP (Innovagen), or 30 µg/ml purified IgG. Erythrocytes were lysed, cells were fixed in 1xPBS/1% PFA, and cytospins were prepared. NET-formation was analyzed by immunofluorescence staining of nuclear and extranuclear DNA with 1 µg/ml DAPI (Invitrogen) and with a 1∶200 dilution of a rabbit anti-neutrophil elastase (NE) antibody (Abcam) and 3.75 µg/ml FITC-conjugated goat anti-rabbit IgG (Jackson ImmunoResearch). Cytospins were examined on a Nikon Eclipse TS 100 microscope. Quantitative morphometrical analysis of NETosis and aggregation of cells was performed on photos of cytospins using Adobe Photoshop CS5 Extended software. To this end, total DAPI-positive events were counted in 3 photos of each treatment condition, and percentages of cells in aggregates >5x mean nuclear size were calculated. Furthermore, NETs (extracellular diffuse DNA colocalizing with neutrophil elastase) were counted and percentages of cells having undergone NETosis were computed.

### Statistical analyses

Within each set of experiments shown in one figure multiple comparisons of groups with a control were adjusted using Dunnet’s or Bonferroni’s post-hoc test. In the case of pairwise comparisons selected *a priori* the method of Bonferroni and Holm was used for adjustment. Adjusted p-values<0.05 were considered to be statistically significant. Levels of significance are labelled throughout the figures as follows: *p<0.05, **p<0.01, ***p<0.001. Computations and charts were performed using GraphPad Prism 5 software.

## Results

### Autoantibodies to LL-37 and raised serum levels of LL-37 but no correlation with disease parameters in SLE

**Figure 1:**
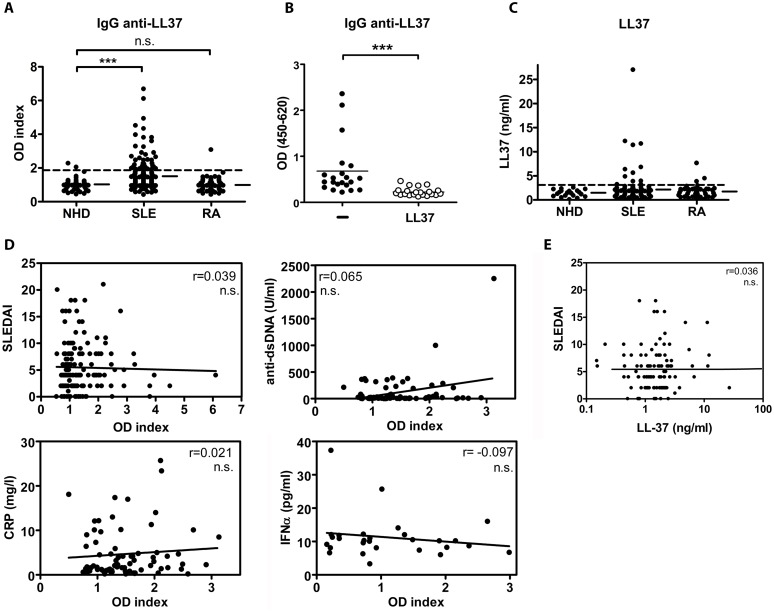
Autoantibodies against LL-37 in SLE are not linked to disease activity. (A) In patients with SLE (n = 185) but not with RA (n = 92) or normal healthy donors (NHD, n = 74) serum autoAbs to LL-37 are detected. Shown are optical density (OD) indices, which are the ratios of the OD in the individual donor to the mean OD in NHD sera. One dot represents one patient, horizontal bars show the means. ***P<0.001, as determined by Kruskal-Wallis test with Dunn’s post-hoc test. Dashed line shows cutoff OD (1,93). (B) Sera of SLE patients (n = 21) were preincubated with saturating concentrations of LL-37 peptide before reactivity to plate-bound LL-37 was determined by ELISA. IgG reactivity to LL-37 can be blocked by preincubation with LL-37 peptide. ***P<0.001, as determined by Mann-Whitney U test. (C) Serum levels of LL-37 peptide are raised in a subgroup of SLE and RA patients. One dot represents one donor. Dashed line shows cutoff OD (2,77 ng/ml). (D) No correlation is found between SLE anti-LL-37 autoAbs and disease parameters of SLE. X-Y scatter plots of correlations between OD indices of anti-LL-37 autoAbs in SLE patients and SLEDAI (n = 163), anti-dsDNA autoAbs (n = 73), or serum levels of C-reactive protein (CRP, n = 74) or of IFNα (n = 28), respectively. Spearman coefficients (r) are depicted within the graphs. N.s., not significant. (E) Serum levels of LL-37 peptide are not linked to SLE disease activity. X-Y scatter plot of correlation between serum LL-37 levels and SLEDAI in 110 patients with SLE. Spearman coefficient (r) is depicted within the graph. N.s., not significant.

**Figure 2:**
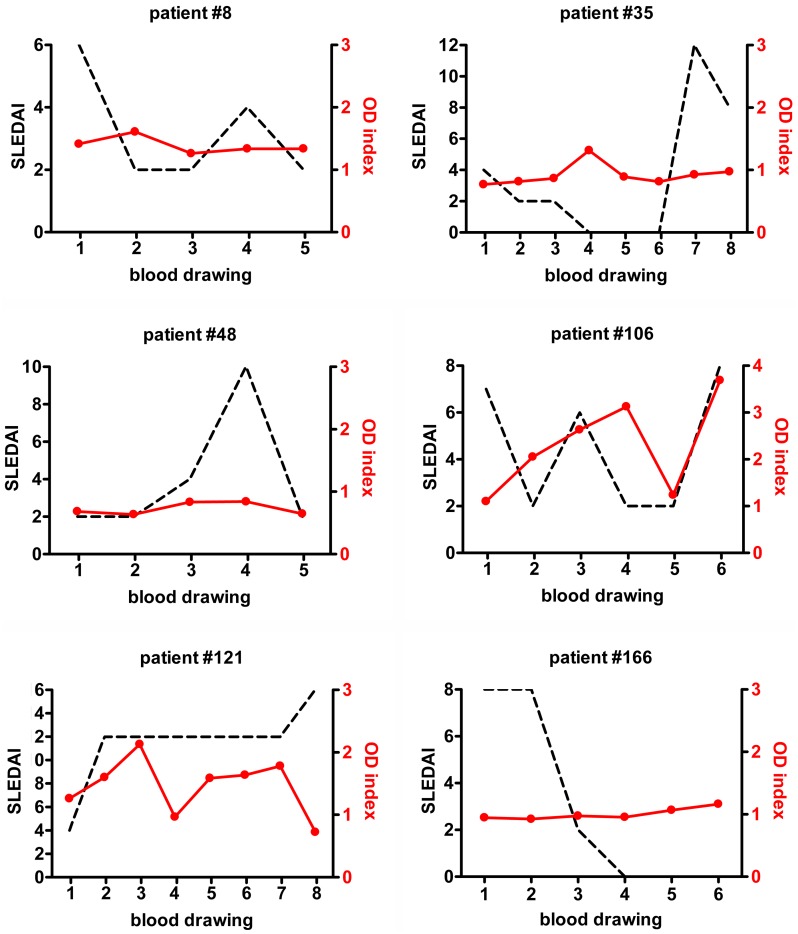
Longitudinal follow up of SLE patients does not uncover an association of anti-LL-37 autoAbs and disease activity. Development over time of SLEDAI (dashed black line) and OD index of anti-LL-37 autoantibodies (red line) in 6 representative patients with SLE. A connection between anti-LL-37 and SLEDAI is not apparent.

We investigated the presence of autoAbs to LL-37 in patient cohorts of SLE and RA originating from Erlangen and Linköping, respectively (S1 Table). We detected significantly higher levels of IgG autoAbs to LL-37 in SLE sera compared to healthy controls (p<0.001) (sensitivity 18,62%, specificity 97,01%), but not in RA (Fig. 1A). Specificity of the anti-LL-37 antibodies was confirmed by inhibition of the reactivity when sera were preincubated with LL-37 (Fig. 1B). Together with anti-LL-37 autoAbs, also serum levels of LL-37 peptide were raised in 13,91% of patients with SLE and in 11,63% of patients with RA but not in sera from healthy donors (Fig. 1C). However, in contrast to previous reports [3], the levels of autoAbs to LL-37 in sera of the two cohorts tested did not correlate to either clinical or laboratory disease parameters of SLE, such as SLEDAI, anti-dsDNA autoAbs, CRP, IFNα (Fig. 1D), or organ involvement (data not shown). Moreover, no correlation was shown in individuals with SLE over the course of the disease between anti-LL-37 and change in SLEDAI (Fig. 2). In line with that, there was no significant correlation of serum levels of LL-37 peptide and disease activity (Fig. 1E), organ involvement, or treatment (not shown). Serum levels of LL-37 peptide did also not correlate with anti-LL-37 autoAbs (not shown).

### No differences in pristane-induced lupus in WT and CRAMP^−/−^ mice

**Figure 3:**
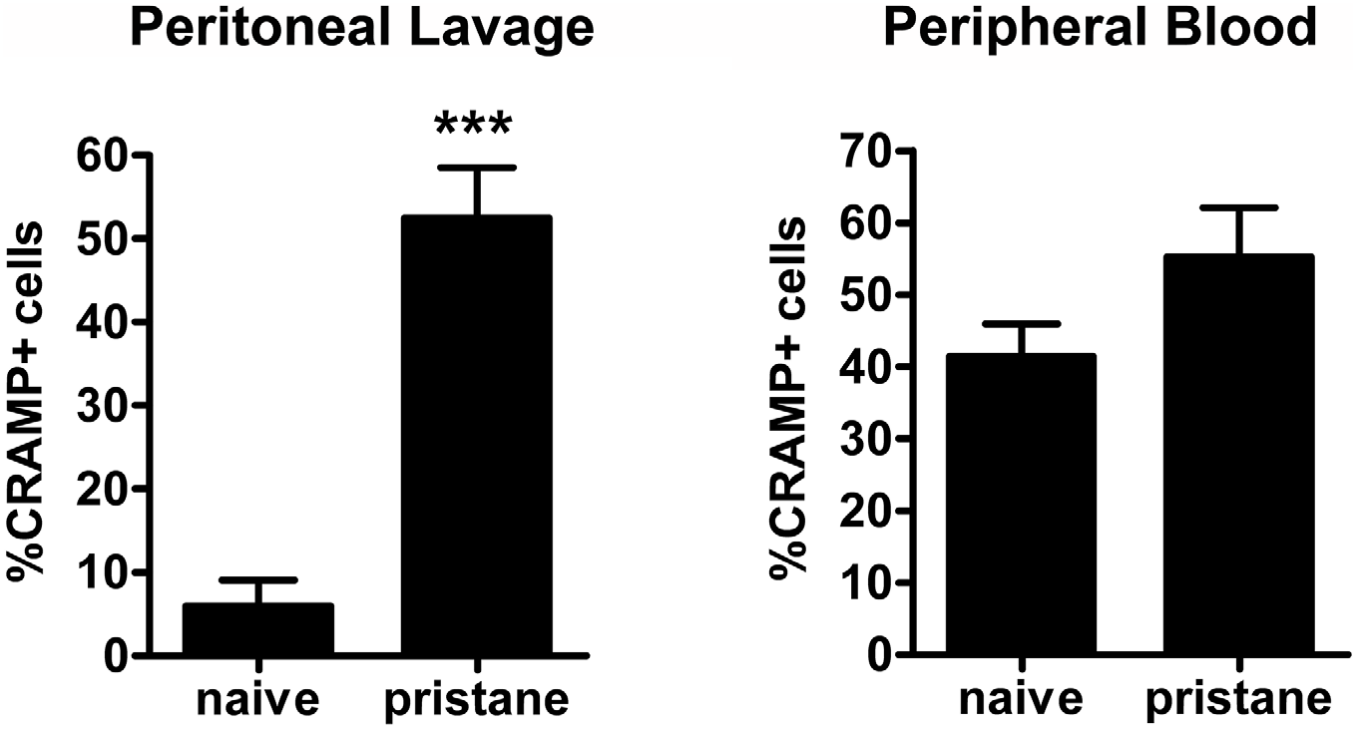
Percentage of CRAMP-expressing cells is higher in pristane-injected animals. Percentage of CRAMP+ cells in peritoneal lavage and peripheral blood of naïve mice and mice 7 days after pristane-injection. N = 5. *P<0.001, as determined by Student’s t-test.

**Figure 4:**
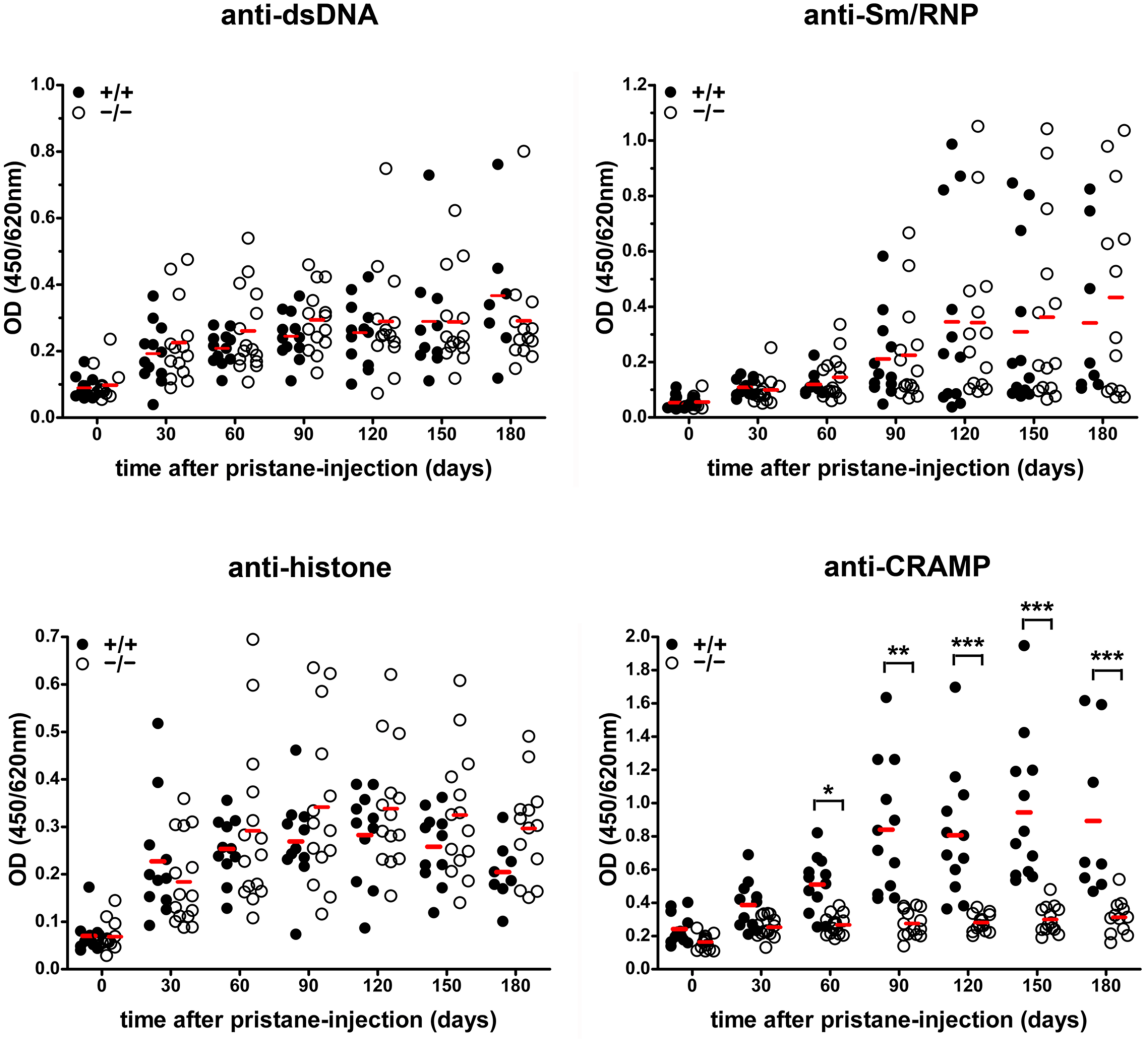
Development of lupus autoAbs in C57BL/6 WT and CRAMP^−/−^ mice. CRAMP^−/−^ and CRAMP^+/+^ mice develop similar levels of lupus autoantibodies after pristane-injection. Autoantibodies to dsDNA, Sm/RNP, histone, and CRAMP were analysed in sera of naïve and pristane-primed mice (n = 11–15) by ELISA and compared by ANOVA with Bonferroni post-hoc test. Red horizontal bars show means. No significant differences could be found except for autoantibodies to CRAMP. *P<0.05, ***P<0.001.

**Figure 5:**
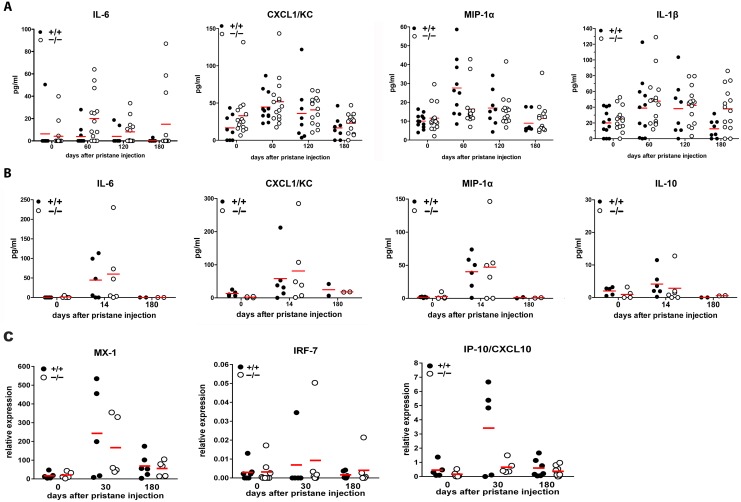
Cytokine/chemokine levels and expression of ISGs in sera and peritoneal lavages of naïve and pristane-injected mice are not significantly influenced by CRAMP-deficiency. Scatter plots show individual measurements and means from naïve and pristane-injected mice of cytokine/chemokine concentrations in serum (A) or peritoneal lavage (B), or of expression of Interferon I-stimulated genes in serum (C), normalized to β-actin expression. One dot represents one mouse. No significant differences were found by ANOVA with Bonferroni post-hoc test.

To investigate the functional involvement of cathelicidins in lupus we injected pristane intraperitoneally into C57BL/6 WT and CRAMP^−/−^ mice and monitored development of autoAbs and organ involvement of PIL over 6 months. The number of CRAMP+ cells in the peritoneum was strongly raised after pristane-injection (Fig. 3). Pristane-injected animals developed typical lupus autoAbs such as anti-dsDNA, anti-Sm/RNP, and anti-histone, but no differences could be observed between CRAMP^−/−^ and CRAMP^+/+^ animals (Fig. 4). CRAMP^+/+^ but not CRAMP^−/−^ mice also developed anti-CRAMP autoAbs. Concentrations of inflammatory mediators were mostly increased in blood and peritoneal lavages after pristane-priming (Fig. 5A and 5B). Again we could not detect significant differences between CRAMP^+/+^ and CRAMP^−/−^ mice. Concentrations of IFNα were below detection limits in both serum and peritoneal lavages (data not shown). Expression of ISGs was mostly raised early after pristane-injection, but there were no significant differences between CRAMP^+/+^ and CRAMP^−/−^ mice (Fig. 5C).

**Figure 6:**
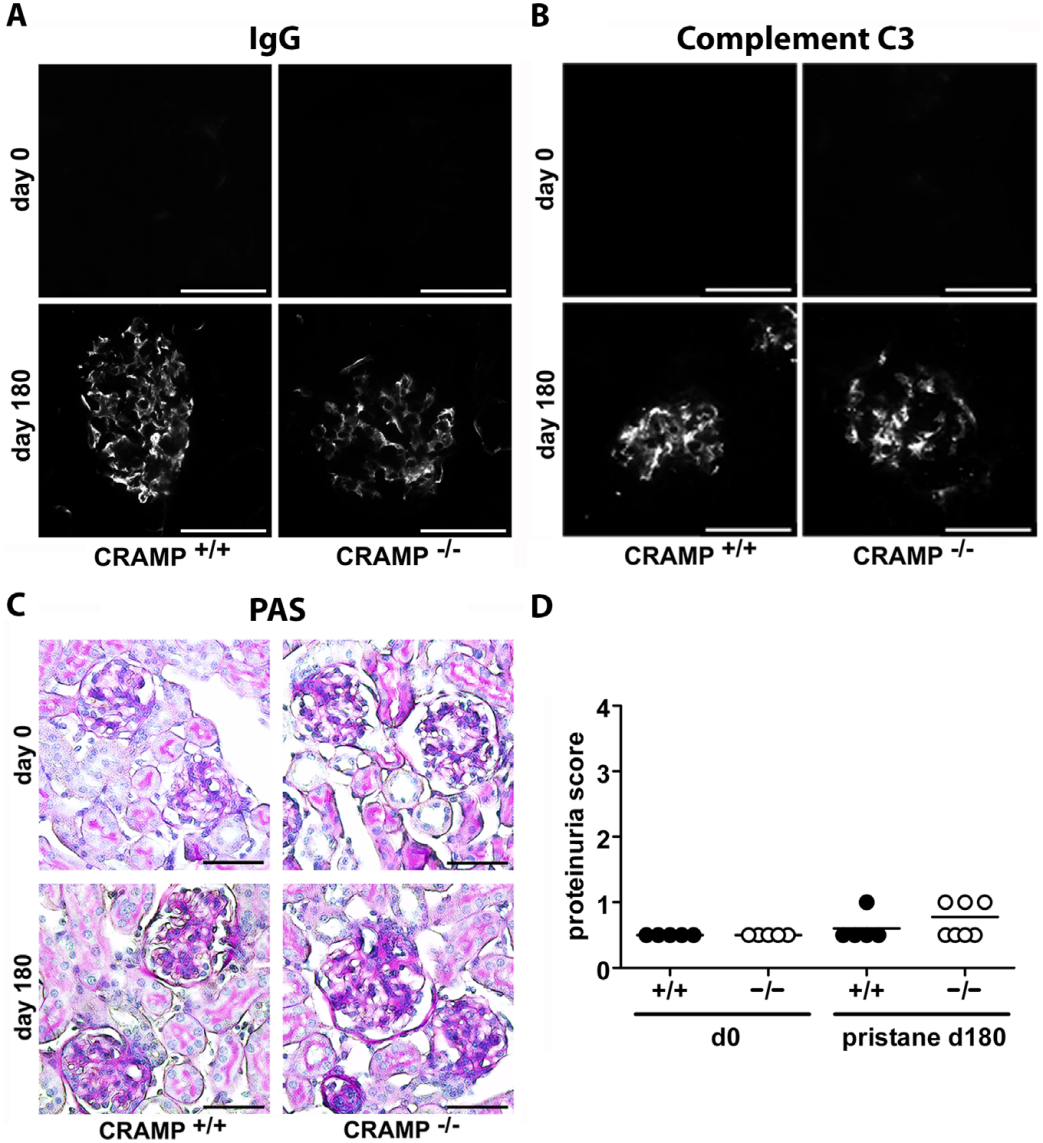
Mild renal disease develops after pristane-injection in both WT andCRAMP^−/−^ mice. Representative immunofluorescence images showing predominantly mesangial IgG-deposits (A) and complement C3 deposits (B) in WT and CRAMP-deficient mice before and 6 months after pristane-injection. Scale bars, 50 µm (C) Representative PAS-stainings of kidneys from unchallenged and pristane-injected WT and CRAMP^−/−^ mice showing mild mesangial cell and matrix expansion at day 180 in both groups. Scale bars, 50 µm (D) Quantification of proteinuria in CRAMP^+/+^ and CRAMP^−/−^ mice before and 180 days after pristane-injection.

**Figure 7:**
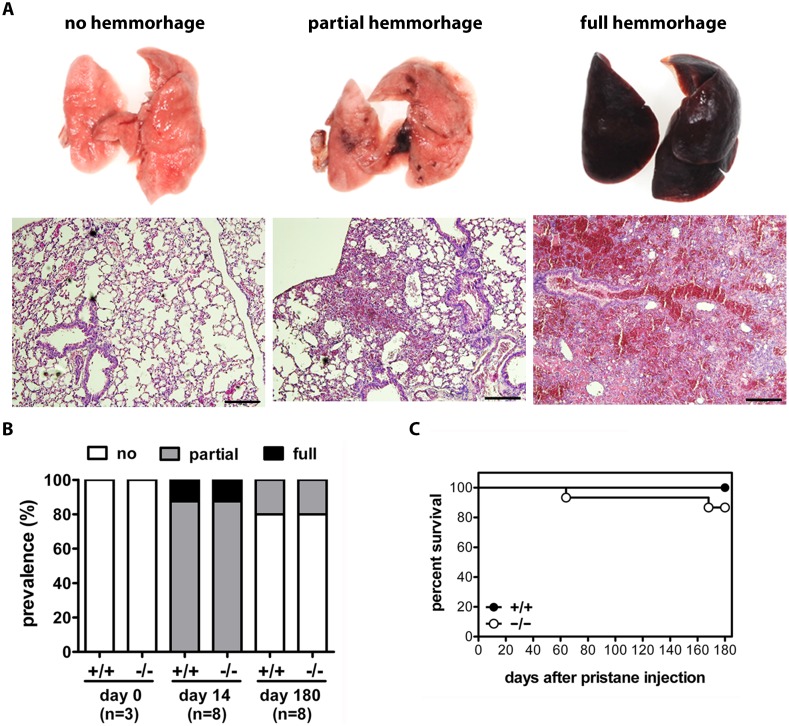
Transient alveolar haemorrhage develops after pristane-injection in both WT and CRAMP^−/−^ mice. (A) Classification of lung pathology in pristane-injected mice. Representative images and H&E-stainings of C57BL/6 mice two weeks after pristane-injection, with lungs showing no hemorrhage, partial, or full hemorrhage. Scale bars, 200 µm. (B) Time course of pristane-induced alveolar hemorrhage in WT and CRAMP^−/−^ mice. Incidence and types of gross pathology at day 0, day 14, and day 180 after pristane-injection. (C) Low mortality after pristane-injection in both CRAMP^+/+^ (n = 14) and CRAMP^−/−^ (n = 15) mice. No significant differences could be found by Logrank test.

As previously reported for mice on the C57BL/6 background [15], [16], we observed only mild renal disease (deposition of complement factor C3 and IgG in the glomeruli with mild mesangial matrix expansion, mild proteinuria, Fig. 6A–D) after i.p. pristane-priming, but found transient hemorrhagic alveolitis (Fig. 7A–C) that, however, only resulted in low mortality (Fig. 7D) in both strains.

### No difference in collagen-induced arthritis in CRAMP^+/+^ and CRAMP^−/−^ mice

**Figure 8:**
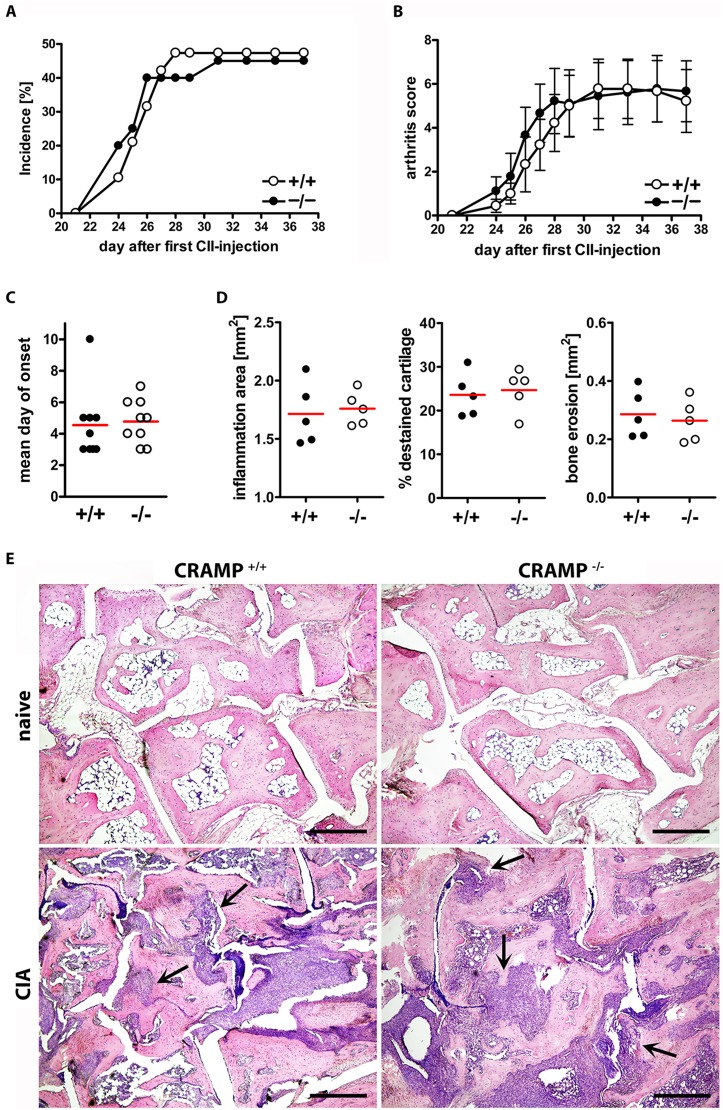
Comparison of collagen-induced arthritis (CIA) in WT and CRAMP-deficient mice. Incidence (A) of CIA and severity in affected mice (B). No significant differences could be found by comparing area under the curves of arthritic WT mice (n = 9) and CRAMP-deficient mice (n = 9) by Student’s t-test. Curves show means ± S.E.M. (C) Onset of CIA is not significantly different in CRAMP^+/+^ and CRAMP^−/−^ mice. (D) Histomorphometric analysis of hind paws from WT and CRAMP^−/−^ mice for synovial inflammation, cartilage damage, and bone erosion. Shown are scatter plots of individual measurements and means. One dot represents one mouse. No significant differences could be found by Student’s t-test. (E) Representative H&E stainings of joint sections from naïve mice and mice 37 days after collagen-injection. Left panels show sections from WT mice, right panels sections from CRAMP-deficient mice. Arrows indicate areas where inflammatory synovial tissue invades the subchondral space. Scale bars, 500 µm.

Cathelicidins are overexpressed during the course of arthritis and targeted by autoAbs and have therefore been implicated in the development of arthritis in humans and rodents [8]. However, so far any evidence about a direct contribution to pathogenesis is missing. To assess the functional involvement of cathelicidins in arthritis, we conducted collagen-induced arthritis (CIA) in C57BL/6 WT and CRAMP-deficient mice. Incidence and severity as well as time of onset of CIA were similar in WT and CRAMP^−/−^ mice (Fig. 8A–C). Histomorphometry revealed no differences between CRAMP^+/+^ and CRAMP^−/−^ mice regarding inflammation area, cartilage degradation, or bone erosions (Fig. 8D–E).

### Functional implications of CRAMP for lupus pathogenesis

The effect LL-37 can have on SLE pathogenesis has been attributed to two modes of action: firstly, LL-37 has been shown to form ionic complexes with nucleic acids that efficiently enter plasmacytoid dendritic cells (pDCs) or monocytes and are retained in early endosomes where they trigger sustained stimulation of TLRs [1], [4]. Secondly, anti-LL37 antibodies were reported to induce formation of neutrophil extracellular traps (NETs). As a consequence of this, the abundant DNA that is present in NETs bound to LL-37 stimulates pDCs to produce Type I IFN [3].

**Figure 9:**
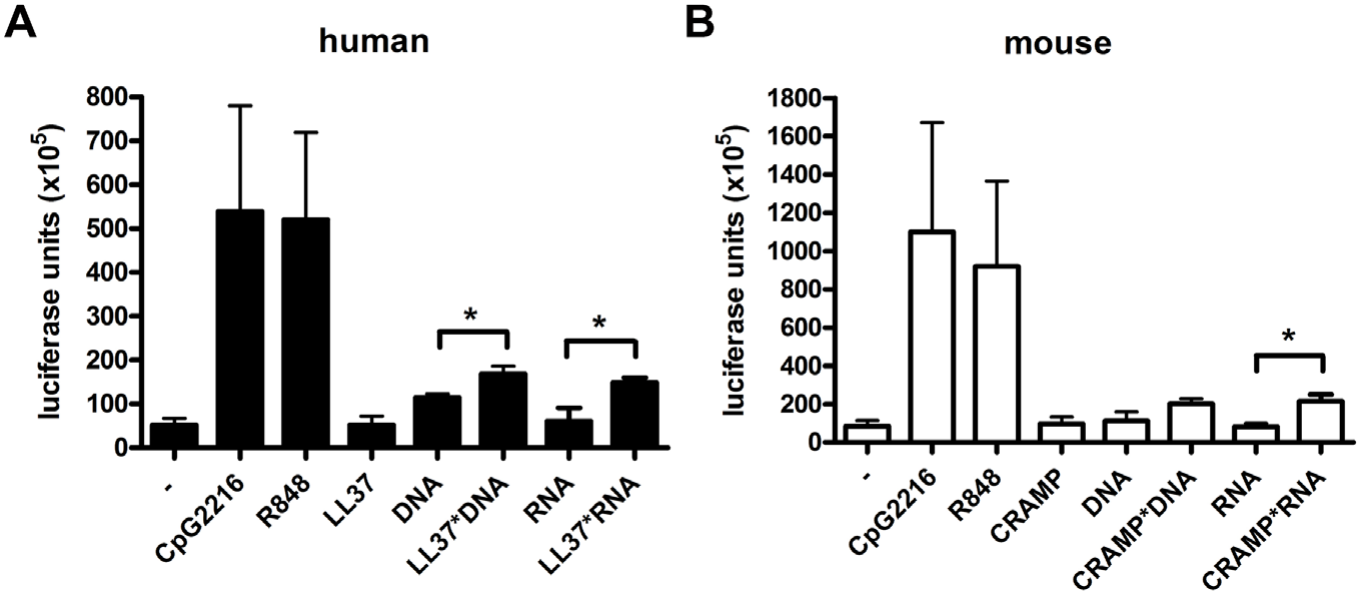
Complexes of cathelicidins and RNA or DNA induce low production of IFNα. Bars show means and SEM of IFNα concentrations in supernatants from isolated human (A) or mouse (B) pDCs after incubation with free genomic DNA, free self-RNA, or DNA/RNA complexed to LL-37 (A) or (CRAMP). IFNα production from unstimulated pDCs or pDCs incubated with LL-37/CRAMP only was determined as a negative control, CpG2216 and R848 (Resiquimod) were used as a positive control for stimulation of TLR9 and TLR7/8, respectively. *p<0.05, as determined by Student’s t-test. N = 4–7.

To determine if similar pathways occur in the murine system, we incubated pDCs isolated from human and mouse blood by MACS with free DNA/RNA or RNA/DNA complexed to CRAMP (Fig. 9). We detected a significant, albeit low induction of IFNα by RNA complexed to both LL-37 and CRAMP, respectively. Complexes with DNA induced a significant induction of IFNα production only in human cells. Considering that in PIL, monocytes have been described as the main producers of IFNα [17] we also used whole blood from WT and CRAMP-deficient mice for our stimulation experiments. However, IFNα production was not significantly enhanced by stimulation with DNA-CRAMP-complexes (data not shown). Thus, in our hands CRAMP shows similar capacity as LL-37 to stimulate IFNα production via complexation to RNA. However, the effect is much lower than previously described [1] and complexation with DNA does not significantly induce IFNα production in mouse cells.

**Figure 10:**
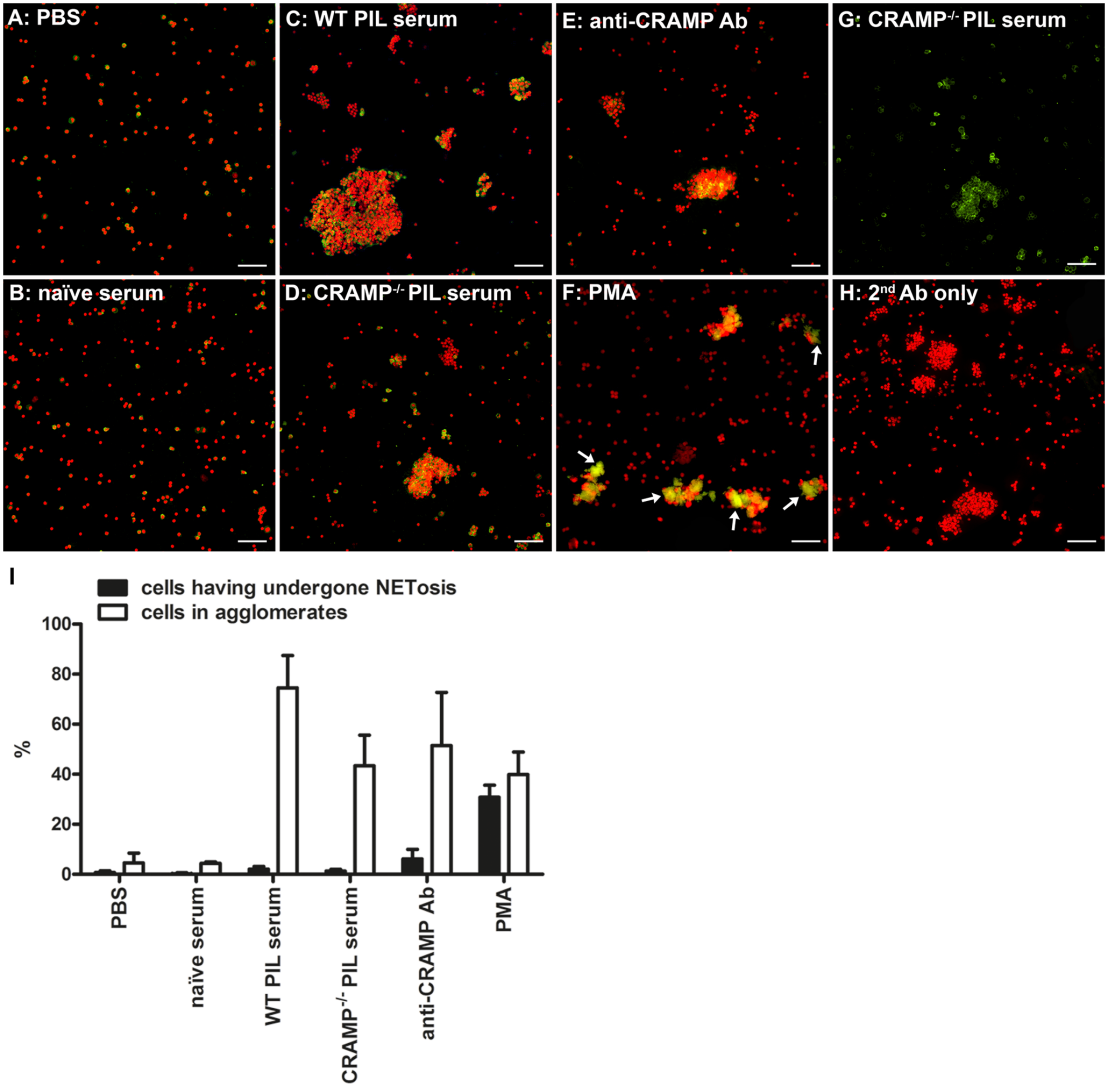
Lupus antibodies induce agglomeration of mouse blood cells but no NETosis. Representative fluorescence microscopy images and morphometry of murine blood cells incubated with various stimuli/controls and stained for DNA (DAPI, red) and neutrophil elastase (green). Incubation with PBS (A), IgG purified from naïve WT mice (B), IgG purified from PIL anti-CRAMP positive sera from WT mice (C), IgG purified from PIL anti-CRAMP-negative sera from CRAMP^−/−^ mice (D), purified anti-CRAMP antibody (E), or PMA (F). The picture in (G) shows only NE-staining and reveals strictly cytoplasmatic expression of NE. Control stainings were performed using secondary FITC-conjugated Ab only (H). Scale bars, 100 µm. Neither incubation with IgG isolated from serum nor incubation with purified anti-CRAMP antibody induced NETosis, identifiable from extracellular washy DNA colocalizing with NE (as shown in F, white arrows). However, IgG from mice with PIL induced agglomeration of blood cells. (I) Quantification of the percentage of cells in aggregates >5x mean nuclear size and of cells having undergone NETosis. Bars show the mean and SD of 3 pictures for each treatment condition.

To address the second described mechanism, we incubated mouse blood from naïve WT mice with IgG isolated from serum of healthy mice and mice with PIL (Fig. 10). After erythrocyte lysis, we prepared cytospins and stained them with a DNA-dye (DAPI) and an antibody to neutrophil elastase (NE). As a control we also used blood cells stimulated with a polyclonal anti-CRAMP antibody, with PMA, a well-known inducer of NETosis, and unstimulated blood cells. Neither incubation with IgG isolated from serum nor with purified anti-CRAMP antibody induced NETosis, identifiable from diffuse extracellular DNA colocalizing with NE [18], [19]. However, IgG from mice with PIL induced agglomeration of blood cells (Fig. 10C & 10D). These agglomerates were induced regardless if the IgG was obtained from anti-CRAMP-positive or -negative PIL serum but not by IgG derived from healthy mice. Also purified anti-CRAMP antibody induced similar aggregates (Fig. 10E). Importantly, these aggregates could be distinguished from NETs because agglomerated cells retained a nuclear localization of DNA and a strictly cytoplasmic expression of NE (Fig. 10G).

## Discussion

Cathelicidins are cationic peptides that are synthesized and stored as inactive precursors in neutrophil granules and are proteolytically maturated before they are released as active AMPs [20], [21]. In humans there is only one cathelicidin gene (*CAMP*) encoding the precursor protein hCAP18 which yields the 37 amino acid long mature peptide LL-37. After being released LL-37 adopts α-helical conformation when interacting with lipid bilayers and anionic compounds [22], [23]. Mice also have only one cathelicidin precursor, which is processed to produce the mature 39-residue α-helical peptide CRAMP [24].

Cathelicidins are an important group of polypeptides released by activated neutrophil granulocytes in inflamed tissues. Neutrophils, long thought to be merely terminally differentiated programmed executor cells, have recently been identified as active participants in triggering and resolving inflammation (reviewed in [25], [26], and [27]). Especially, their ability to release NETs has been in the focus of many interesting studies. In lupus and arthritis, the DNA and attached proteins (such as AMPs) have been suggested to serve as source for the development of autoreactivity [2], [3], [8].

In this study, we investigated autoreactivity to cathelicidins and their direct contribution to lupus and arthritis pathogenesis. Although we found autoAbs in lupus against cathelicidins, we could not detect a functional correlation to disease activity such as SLEDAI and other disease parameters. Furthermore, experimental lupus was not inhibited in the absence of cathelicidins, although CRAMP^−/−^ mice were able to form NETs (not shown).

For our studies, we used the PIL mouse model that is induced by i.p. injection of the isoprenoid alkane pristane and is mainly driven by TLR7-mediated production of type I IFN by inflammatory monocytes [28]. The exact mechanism of action of pristane has yet not been fully elucidated, but it is known that it induces massive cell death [29], [30]. In WT mice, RNA released from dying cells can then bind to CRAMP locally released from infiltrating neutrophils or other cell types. These complexes have been shown to gain access to pDCs and to monocytes [1], [4], [31] in humans and trigger prolonged type I IFN production. In contrast, in SLE cathelicidins were suggested to contribute to pathogenesis by shuttling DNA into pDCs and triggering prolonged stimulation of TLR9. Therefore, although we do not claim that PIL represents all aspects of SLE in humans, we consider it a useful model for addressing the importance of cathelicidins for lupus pathogenesis. However, we cannot exclude a role of cathelicidins in genetical lupus models such as NZB/W F1 or MRL/*lpr* mice.

From our results we deduct that autoreactivity against cathelicidins does not seem to be indispensable for lupus and arthritis pathogenesis. However, in humans other cationic AMPs such as the β-defensins hBD2 and 3 and lysozyme have recently been shown to possess strong binding capacity of nucleic acid and stimulate pDC-activation and inflammation in a way similar to cathelicidins [32], [33]. Thus, whether autoreactivity against exposed cathelicidins is an epiphenomenon due to extensive disease-induced overexpression, or if these AMPs could potentially compensate for the absence of cathelicidins is a question that merits further studies.

## Supporting Information

S1 Table **Serologic and clinical parameters and current treatment of individuals with SLE enrolled in the study.**
(XLS)

S1 Compressed/ZIP File Archive **Raw data of** **Figs. 1** **to** **10** **.**
(ZIP)
